# Long-read genome sequencing identifies cryptic structural variants in congenital aniridia cases

**DOI:** 10.1186/s40246-023-00490-8

**Published:** 2023-06-02

**Authors:** Alejandra Damián, Gonzalo Núñez-Moreno, Claire Jubin, Alejandra Tamayo, Marta Rodríguez de Alba, Cristina Villaverde, Cédric Fund, Marc Delépine, Aurélie Leduc, Jean François Deleuze, Pablo Mínguez, Carmen Ayuso, Marta Corton

**Affiliations:** 1 Department of Genetics & Genomics, Instituto de Investigación Sanitaria-Fundación Jiménez Díaz University Hospital, Universidad Autónoma de Madrid (IIS-FJD, UAM), 28040 Madrid, Spain; 2 Centre for Biomedical Network Research On Rare Diseases (CIBERER), 28029 Madrid, Spain; 3 Bioinformatics Unit, Instituto de Investigación Sanitaria-Fundación Jiménez Díaz University Hospital, Universidad Autónoma de Madrid (IIS-FJD, UAM), 28040 Madrid, Spain; 4 Centre National de Recherche en Génomique Humaine, Université Paris-Saclay, 91057 Evry, France; 5 Department of Surgery, Medical and Social Sciences, Faculty of Medicine and Health Sciences, Science and Technology Campus, University of Alcalá, 28871 Alcalá de Henares, Spain

**Keywords:** *PAX6*, Aniridia, Long-read genome sequencing, Nanopore sequencing, Chromosomal rearrangements

## Abstract

**Background:**

Haploinsufficiency of the transcription factor PAX6 is the main cause of congenital aniridia, a genetic disorder characterized by iris and foveal hypoplasia. 11p13 microdeletions altering *PAX6* or its downstream regulatory region (DRR) are present in about 25% of patients; however, only a few complex rearrangements have been described to date. Here, we performed nanopore-based whole-genome sequencing to assess the presence of cryptic structural variants (SVs) on the only two unsolved “*PAX6*-negative” cases from a cohort of 110 patients with congenital aniridia after unsuccessfully short-read sequencing approaches.

**Results:**

Long-read sequencing (LRS) unveiled balanced chromosomal rearrangements affecting the *PAX6 locus* at 11p13 in these two patients and allowed nucleotide-level breakpoint analysis. First, we identified a cryptic 4.9 Mb de novo inversion disrupting intron 7 of *PAX6,* further verified by targeted polymerase chain reaction amplification and sequencing and FISH-based cytogenetic analysis*.* Furthermore, LRS was decisive in correctly mapping a t(6;11) balanced translocation cytogenetically detected in a second proband with congenital aniridia and considered non-causal 15 years ago. LRS resolved that the breakpoint on chromosome 11 was indeed located at 11p13, disrupting the DNase I hypersensitive site 2 enhancer within the DRR of *PAX6,* 161 Kb from the causal gene. Patient-derived RNA expression analysis demonstrated *PAX6* haploinsufficiency, thus supporting that the 11p13 breakpoint led to a positional effect by cleaving crucial enhancers for *PAX6* transactivation. LRS analysis was also critical for mapping the exact breakpoint on chromosome 6 to the highly repetitive centromeric region at 6p11.1.

**Conclusions:**

In both cases, the LRS-based identified SVs have been deemed the hidden pathogenic cause of congenital aniridia. Our study underscores the limitations of traditional short-read sequencing in uncovering pathogenic SVs affecting low-complexity regions of the genome and the value of LRS in providing insight into hidden sources of variation in rare genetic diseases.

**Supplementary Information:**

The online version contains supplementary material available at 10.1186/s40246-023-00490-8.

## Background

Aniridia (MIM #106,210) is a rare Mendelian panocular disease with an estimated worldwide incidence of 1:80,000 births, characterized primarily by a combination of iris and foveal hypoplasia [1]. Aniridia is a highly penetrant autosomal dominant disease caused almost exclusively by haploinsufficiency of *PAX6* (*Paired box gene 6*) [2], a transcription factor essential for ocular development that regulates highly complex gene networks [3]. The correct dosage and fine-tuned spatiotemporal expression of *PAX6* are tightly controlled by more than 30 cis-regulatory elements [4], most of which are found up to 150 Kb of the *PAX6* coding sequence in the so-called “Downstream Regulatory Region” (DRR) [2, 3].

Structural variants (SVs) on chromosome 11p13 play a crucial role in 25–30% of patients with congenital aniridia by affecting *PAX6* expression [2]. These mainly include microdeletions spanning the *PAX6* genomic sequences or only the distant DRR [2]. Contiguous gene deletions of *PAX6* and neighboring genes, such as *WT1*, are involved in a syndromic form of aniridia characterized by Wilms tumor, Aniridia, Genitourinary anomalies, and mental Retardation called WAGR syndrome [5]. However, except for copy number variations (CNVs), hardly any other chromosomal rearrangements have been reported in patients with congenital aniridia, mainly chromosomal translocations with 11p13 breakpoints affecting *PAX6* [6–8].

Nowadays, pathogenic *PAX6* variants are identified in ~90% of patients with classical aniridia [9]. Recent advances in genomic sequencing have identified new molecular mechanisms underlying the etiopathogenesis of congenital aniridia involving non-coding variants [10, 11]. However, few cases remain unsolved after having exhaustively ruled out intragenic *PAX6* mutations and 11p13 microdeletions, or even other rare variants in some aniridia-mimicking genes of anterior segment dysgenesis, such as *ITPR1, FOXC1, PITX2, or CPAMD8* [12–14]. Although other disease-associated *loci* may remain to be discovered in congenital aniridia, *PAX6* inversions or other complex SVs may have been missed during genetic analysis in unsolved cases.

Short-read next-generation sequencing (NGS) and traditional cytogenetic approaches currently used to identify disease-causing variants in clinical routine have low resolution or technical limitations in revealing SVs in highly repetitive regions or involving no apparent loss or gain of genetic material [15, 16]. Short-read whole-genome sequencing (WGS) can detect balanced SVs more efficiently than targeted NGS approaches [16], but also has some shortcomings for sequencing low-complexity regions, thus, resulting in sequence gaps and incomplete assemblies that may mask the presence of certain types of SVs [17, 18].

Emerging long-read sequencing (LRS) technologies have great potential to overcome these limitations by producing read lengths of several kilobases, thus creating complete overlaps that allow the generation of high-quality genomic assemblies without altering its original linear genomic structure [17, 18]. Recently, different studies have shown that combining LRS and cytogenetic tools increases the detection rates of these underrated SVs in different genetic diseases [19–22].

Here, we aimed to evaluate whether genetically uncharacterized cases with congenital aniridia would carry hidden balanced SVs affecting *PAX6* or its regulatory elements. We present the study of two unsolved sporadic cases from a large cohort of patients with congenital aniridia, in which nanopore-based LRS uncovered chromosomal breakpoints within the 11p13 region, previously masked in short-read NGS and cytogenetic approaches. Therefore, our work highlights the utility of new long-read WGS to successfully identify the missing genetic cause in “*PAX6*-negative” cases.

## Results

Two sporadic patients (ANI-1 and ANI-2) suffering from congenital aniridia without a conclusive genetic diagnosis were enrolled at the Fundación Jiménez Díaz University Hospital (Madrid, Spain), as part of a large study intended to unveil SVs in rare eye diseases using nanopore-based LRS. To elucidate the genetic etiology, both cases had previously been screened by Sanger sequencing, karyotyping, and multiplex ligation-dependent probe amplification (MLPA) assays using routine testing for congenital aniridia [23]. As second-line tests, the complete *PAX6 locus* and its distant regulatory regions were thoroughly screened by custom chromosomal microarray analysis (CMA) and short-read NGS, either by targeted gene panel or WGS [5, 10]. However, all these analyses failed to uncover a likely molecular diagnosis, as no potential causative exonic or non-coding variants, loss (or gain) of genetic material, and/or apparent chromosomal rearrangement at the *PAX6* locus were detected [5, 10].

Using the PromethION platform from Oxford Nanopore Technologies, a long-read WGS was performed to analyze potential SVs affecting *PAX6* (Additional file 1: Figure S1). An average sequence depth of 43 × was achieved with a median read length of 9971 bp and 4800 bp for ANI-1 and ANI-2, respectively (Additional file 1: Table S1). Over 99% of the reads aligned to the human reference genome (GRCh38). Additional sequencing and mapping quality data are shown in Additional file 1: Table S1 and Figure S2. In both patients, Sniffles2 [24, 25] SV calling identified an SV in which at least one breakpoint was located at 11p13, disrupting the *PAX6 locus* (Table 1).

**Table 1:** Summary of the phenotypic and genomic findings of the two cases

Sample	Phenotype	Type	Zygosity	Depth read (DR/DV)	Cytoband	Genomic Position (GRCh38)	Target gene
ANI-1	Iris hypoplasia, congenital nystagmus, cataracts, PHVP	Inversion	HET	17/47	11p13	11:31,795,178	*PAX6 *(intron 7)
					11p12	11:36,791,763	Intergenic region
ANI-2	Aniridia (RE), iris coloboma (LE), congenital cataracts	Translocation	HET	8/20	11p13	11:31,656,015	*ELP4* (HS2-*PAX6)*
					6p11.1	6:59,055,694	Centromeric region (alpha-satellite DNA)

PHPV: Persistent hyperplastic primary vitreous, RE: right eye, LE: left eye, HS2: DNaseI hypersensitive site 2. DR: high-quality reference reads, DV: high-quality variant reads

### LRS identified a cryptic inversion at 11p13

The first patient (ANI-1) was a girl referred for genetic testing at 12 years of age with a clinical diagnosis of congenital aniridia. She was born to a healthy non-consanguineous couple with no family history of eye disorders and had two unaffected siblings. Since birth, she presented bilaterally with severe iris hypoplasia, cataract, nystagmus, and persistent hyperplastic primary vitreous (PHPV) (Table 1). She had normal development without any other extraocular symptoms. Despite successive *PAX6* genetic testing over the years, the patient remained genetically undiagnosed.

**Fig. 1:**
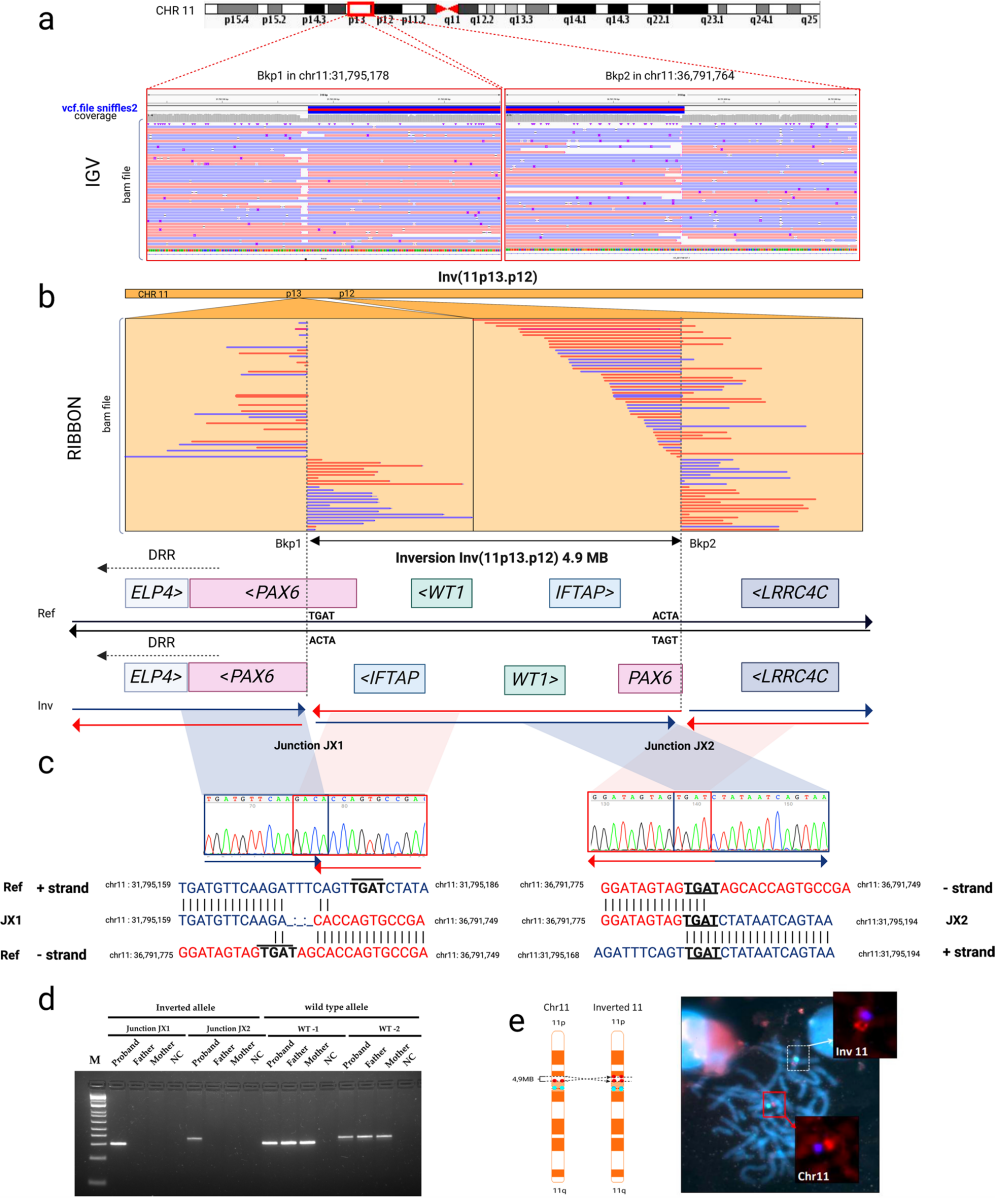
Identification and validation of a balanced paracentric inversion involving *PAX6* in patient ANI-1. ***a–b.*** Representation of long reads, breakpoints, and genomic elements involved in a 4.9 Mb paracentric inversion on chromosome 11. Nanopore long-read genome sequencing from high molecular weight DNA revealed a cryptic heterozygous inversion disrupting *PAX6*. The IGV and Ribbon tools were used to visualize read alignments from vcf and BAM files**.** Genes and regulatory elements most relevant to phenotypic expression of congenital aniridia, including *PAX6* and downstream regulatory regions (DRR) and *WT1* and adjacent genes next to the breakpoints, are depicted. BKP1 and BKP2: distal and proximal breakpoints. **c.** Validation of the distal breakpoint of the inverted allele fragment junctions (JX1 and JX2) on both sides by PCR and Sanger sequencing. **d.** Electrophoresis of the amplified-fragment junctions in the proband showed unique bands corresponding to the inverted allele in JX1 and JX2, while amplicons for the wild-type allele were also present in both parents. NTC: no template control. **e.** Schematic representation of the inversion and FISH findings. Centromeric D11Z1 and *PAX6* probes are labeled in cyan blue and red, respectively. FISH analysis showed a well-defined red-associated *PAX6* signal on wild-type chromosome 11 and spread and separated red-associated *PAX6* signals on the inverted chromosome

LRS analysis uncovered a 4.9 Mb heterozygous paracentric inversion at 11p13-11p12 spanning 49 known genes, including *PAX6* and *WT1* (Table 1). LRS also allowed fine mapping of the two breakpoints (Fig. 1a). The distal breakpoint (BKP1) on 11p13 is located at chr11:31,795,178 (hg38), disrupting intron 7 of the canonical *PAX6* isoform NM_000280.5 (Fig. 1a and Additional file 1: Fig. 3). Thus, the promoter region and the first 6 out of 13 exons of *PAX6* are taken apart from the 3’ gene region and the distant cluster of cis-regulatory elements located in the DRR of *PAX6* (Fig. 1b). The proximal breakpoint (BKP2) lies at chr11:36,791,763 (hg38) in an intergenic region at 11p12; thus, it does not disrupt any known genes.

To validate the inversion and its breakpoints at the single nucleotide level, a PCR-based validation assay was specifically designed to amplify the junction sequences of the inverted allele (Additional file 1: Table S2). Both breakpoint junctions called by Sniffles2 were consistent with those obtained by Sanger sequencing (Fig. 1c). This strategy also confirmed that this inversion occurred de novo in the proband, as the sequences for the inverted allele were not amplified in either parent (Fig. 1d). Inspection of both breakpoint sequences revealed a small 3-bp deletion in BKP1 and 4-bp microhomology region (TGAT) on both sides (Fig. 1c), suggesting that these sequence signatures arose from a potential microhomology-mediated mechanism of inversion formation. Finally, inversion was also confirmed cytogenetically by a fluorescence in situ hybridization (FISH) assay using *PAX6* and centromeric-specific probes for chromosome 11 labeling (Fig. 1e). Scattered signals from the *PAX6* probe observed on the inverted chromosome confirmed the disruption of this gene (Fig. 1e).

### LRS resolved a complex translocation t(6,11) with cryptic breakpoints leading to a positional effect of *PAX6*

The second patient (ANI-2) was a girl referred for genetic testing due to the presence of congenital aniridia, iris coloboma, and congenital cataracts at 2 years of age, following the finding of an apparently balanced de novo reciprocal translocation between the short arms of chromosomes 6 and 11 [46,XX,t(6;11)(p21;p15.5)dn]. This SV was distant from the *PAX6*-associated 11p13 *locus* and was therefore considered non-causative at that time. Subsequently, CMA using the high-resolution custom WAGR array did not identify any gains or losses affecting the *PAX6 locus* or chromosome 11, as previously reported [5]. Since Sanger sequencing-based *PAX6* screening ruled out any disease-causing SNVs, we performed short-read WGS, achieving an average sequence depth of ∼30x, to accurately map translocation breakpoints. Unfortunately, this analysis was also uninformative, as no potential pathogenic SV junctions or split reads were found after SV calling or visual inspection of read alignments at 6p21 and 11p15.5.

**Fig. 2:**
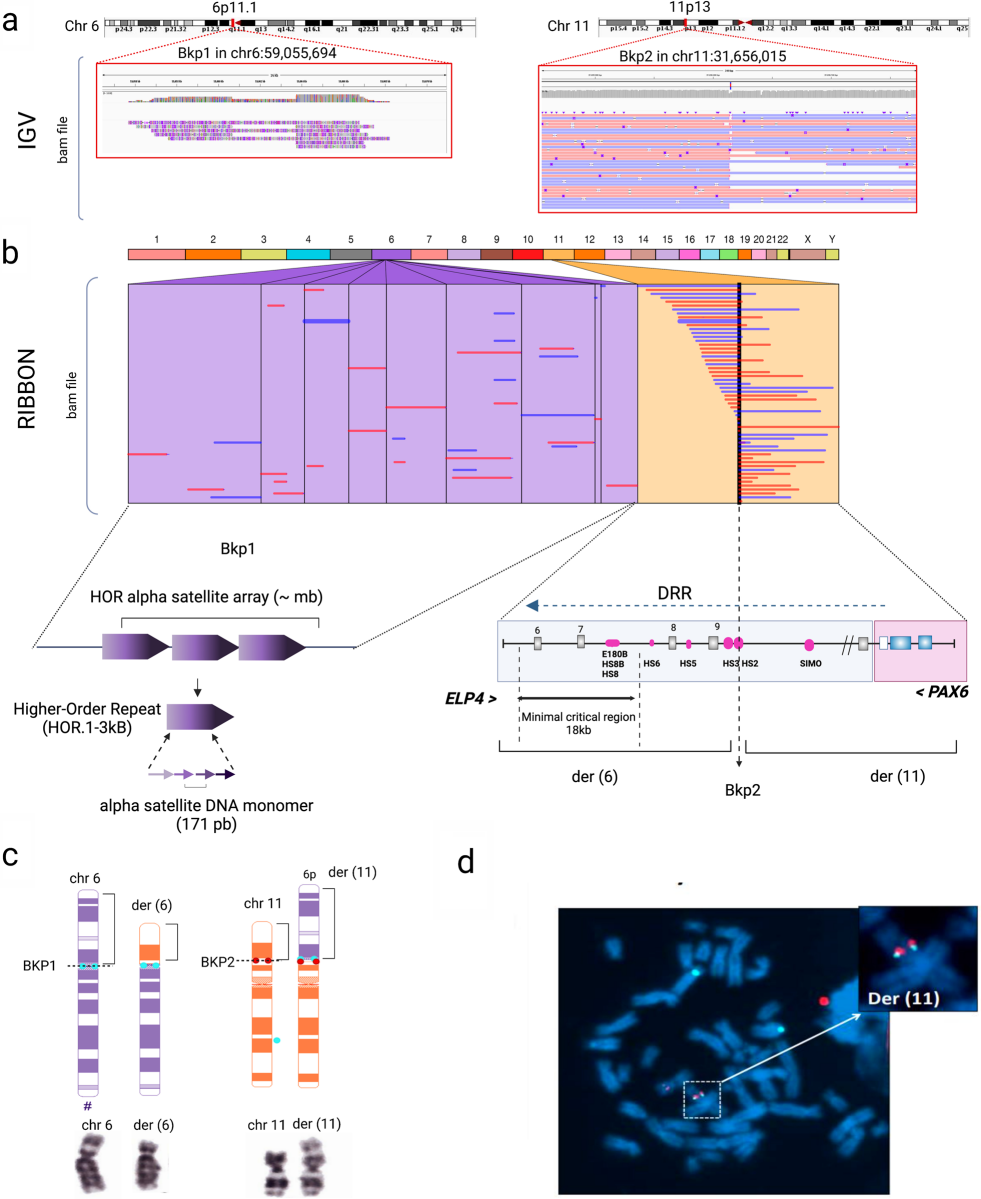
Identification of a reciprocal translocation t(6;11) affecting the *PAX6* downstream regulatory region in patient ANI-2. **a–b**. Representation of long-reads, breakpoints, and genomic elements involved in the paracentric inversion using IGV and Ribbon visualizers from vcf and BAM files. LRS showed evidence of an interchromosomal translocation involving alpha-satellite DNA sequences from the centromeric region of chromosome 6 (6p11.1) and their mate split reads laying on chromosome 11 (11p13) in intron 9 of the *ELP4* gene. The breakpoint on 11p13 disrupts the DNase I hypersensitive site 2 (HS2) enhancer within the downstream regulatory region (DRR) of *PAX6*. This chromosomal break is taking apart *PAX6* from its critical 18 Kb minimal regulatory region defined by Plaisancie et al., 2018 [10]. **c–d.** Karyotyping and FISH analysis showed the presence of a t(6;11) reciprocal translocation. Schematic representation of the FISH assay designed to visualize the breakpoints. The centromeric CEP6-D6Z1 and *PAX6* probes are labeled in cyan blue and red, respectively. FISH images showed specific signals for *PAX6* and CEP6-D6Z1 alpha-satellite sequences on derivative chromosome 11, der (11). A signal for CEP6-D6Z1 was also observed on derivative chromosome 6, der(6), confirming breakpoint involvement at the centromeric level

Finally, Sniffles2-based SV calling from long-read WGS data identified 20 chimeric read sequences mapped on chromosomes 6 and 11, as shown in Fig. 2a-b and Additional file 1: Fig. S4. On chromosome 6, these chimeric reads showed variable homology with different junctions mapped along the centromeric region at 6p11.1 (Fig. 2a-b). Due to the highly repetitive nature of the centromeric-associated alpha-satellite DNA, it was not easy to determine the exact position of the breakpoint at 6p11.1. However, we were able to define a more precise approximate position at chr6:59,055,694 (hg38) according to Sniffles2 SV calling.

The second translocation breakpoint was accurately defined by chimeric reads mapping to 11p13, corresponding to position chr11:31,656,015 (hg38, Table 1). This junction was located in intron 9 of the canonical *NM_019040.5* isoform of the *ELP4* gene (Fig. 2b), located approximately 161 kb from the polyadenylation site of *PAX6*, but within the DRR cluster of *PAX6* enhancers. The breakpoint completely disrupted a *PAX6*-associated regulatory element, the DNase I hypersensitive site (HS2) (Fig. 2b). Although we assessed the breakpoint sequences to understand the possible mechanism leading to the translocation, no microhomologies or repetitive elements were identified at 11p13.

Given the highly repeat-rich region of centromeric sequences involved in the translocation, PCR confirmation of the breakpoints could not be performed. However, LRS findings were confirmed through FISH analysis using a *PAX6-*specific probe (11p13) and a centromeric-specific probe for chromosome 6 (D6Z1), respectively. Normal signals were found on chromosomes 6 and 11 and derivative chromosome 6 (Fig. 2d). In contrast, colocalization of both signals for *PAX6* and CEP6 was found on derivative chromosome 11 (Fig. 2d), thus confirming the presence of centromeric sequences from chromosome 6. According to the results obtained with all the different techniques used, the breakpoints involved in the translocation were redefined, resulting in t(6;11)(p11.1;p11.3) (Fig. 2c).

### Characterization of the molecular pathogenesis of the SVs affecting 11p13

**Fig. 3:**
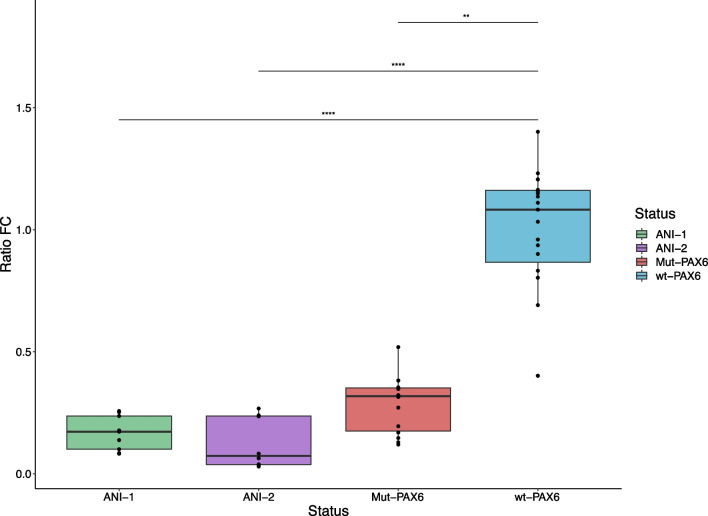
Functional characterization of SVs affecting PAX6 expression. Boxplots represent the relative *PAX6* mRNA expression in a lymphocyte cell line derived from both SV-carrying probands compared to three healthy controls (wt-*PAX6*) and three patients carrying *PAX6*-associated aniridia pathogenic variants from our cohort. *RPLP0* gene was used for setting relative expression. The experiment was performed in triplicate. Boxplots indicate median values and range of whiskers variation. FC: Fold-change indicating the normalized ratio of *PAX6/RPLP0* expression levels. Statistical analysis was performed using Kruskal–Wallis test with Dunn's test for multiple comparisons of normalized *PAX6* expression ratios and Bonferroni for multiple testing corrections. Significance was defined as **p.adj < 0.01, ****p.adj < 0.0001, and p.adj > 0.05 (not significant, ns)

To further explore the possibility of *PAX6* transcriptional deregulation caused by both SVs, we directly assessed RNA expression by specific Taqman-based ddPCR assays in lymphocyte cell lines (LCLs) derived from the two patients. Compared to two unrelated controls without aniridia (Fig. 3), both patients showed a significant reduction in normalized expression of *PAX6* using *RPLP0* as housekeeping gene (ANI-1, fold-change (FC) = 0.017; Dunn's test: adjusted p-value = 1.560E-05; ANI-2, FC = 0.073; p = 1.49E-06), and also a slight statistically non-significant reduction to those observed in two other patients from our cohort carrying disease-causing variants affecting *PAX6* splicing (Additional file 1: Table S3).

## Discussion

Chromosomal breakpoints can alter the linear structure of a gene, but also lead to positional effects by separating distant cis-regulatory elements from the causative gene body, thus resulting in defective expression [26]. Three decades ago, some studies first elucidated the hypothesis of positional effects underlying SVs in congenital aniridia by cytogenetic assays [27, 28]. Since then, only a few cases carrying chromosomal inversions or translocations have been reported to date, and in none of them the precise breakpoint positions have been pinpointed [6–8]. Here, we speculate that hidden SVs at the *PAX6 locus* might have been overlooked due to limitations of cytogenetic or short-read NGS technologies and, consequently, might partly explain some of the unsolved cases with congenital aniridia that remain to be genetically characterized. In our own experience, NGS has efficiently increased the genetic yield of congenital aniridia to > 90%, as other authors also reported [29]. However, there are still patients with a clinical diagnosis of congenital aniridia for whom the genetic cause remains elusive.

Third-generation LRS sequencing allowed us to unravel the only two unsolved cases of congenital aniridia in our cohort, for which different genetic testing approaches had been sequentially performed for > 10 years unsuccessfully. Following nanopore-based WGS and RNA analysis, we identified two SVs that reduce *PAX6* expression by directly disrupting the gene structure or a distant enhancer in its downstream regulatory region. In both cases, these molecularly proven pathogenic SVs had remained undetected by short-read sequencing.

In the first case, a 4.9-Mb hidden balanced paracentric inversion was detected with its distal breakpoint on intron 7 of *PAX6*. Prior to being analyzed by LRS, short-read data was available from an amplicon-based capture strategy aimed at capturing the complete *PAX6* gene [10], whose CNV analysis had suggested the presence of a small 450 bp deletion within this same intron (chr11:31,794,990–31,795,440, Additional file 1: Fig. S5). Given the low confidence in CNV calling, we rule this out as a probable artifact. Supporting this, no microdeletions affecting the intron 9 were detected by high-resolution custom CMA targeting the *PAX6 locus* [10], as ~50 non-overlapping 60-mer probes covered this 6.3-Kb region well. Following LRS findings, we speculate whether this anomalous call was indeed indicative of the presence of a breakpoint junction in this intron or whether it was coincidental. LRS revealed that this inversion is a balanced event, so, in principle, read-depth-based CNV analysis would be missed it. However, the breakpoint overlapped with 7 of the circular amplicons designed to capture this region (Additional file 1: Fig. S5). Therefore, it is plausible that the poor coverage could have arisen due to these technical reasons, leading to an artefactual deletion call. Besides, we cannot rule out that this inversion would have been unraveled by short-read WGS in case the distal breakpoint located in an intergenic region had been sequenced with sufficient coverage. This inversion also remained hidden in a previous karyotyping analysis, as it was below the detection threshold of this technique. But, a further FISH analysis designed specifically to target *PAX6* confirmed the disruption of this *locus.*

Thanks to LRS analysis, we concluded that a chromosomal translocation initially ruled out as causative is solely responsible for the congenital aniridia presented in the second patient. About 15 years ago, a karyotyping analysis revealed a presumably non-causal de novo reciprocal t(6;11) translocation that appeared to affect the telomeric cytoband 11p15.5, far away from the aniridia-related 11p13 *locus*. Despite our efforts to investigate the genetic cause of aniridia using sequentially all emerging techniques at our disposal [5, 10], this patient remained unsolved after almost two decades of study. After ruling out all possible pathogenic variants, we finally hypothesized that this translocation might be causal but was not correctly defined. To do so, we performed a short-read WGS analysis that, unexpectedly, did not allow us to resolve the breakpoints on any of the chromosomes involved. This failed analysis could indicate that the junctions encompassed highly repetitive regions that were missed by short-read sequencing. Indeed, only after nanopore-based WGS analysis we were able to fully determine the thus-far overlooked exact breakpoints of the t(6,11) on both chromosomes.

On chromosome 11, the breakpoint is located at the aniridia-associated 11p13 *locus* affecting the DRR cluster of *PAX6* downstream cis-regulatory elements. Fine mapping of the breakpoint sequences revealed that this junction disrupts a retinal enhancer called HS2, whose deletion has been reported to affect *PAX6* expression in the neural retina leading to abnormal eye development [30]. Therefore, our work confirms the importance of the sequence integrity of *PAX6* enhancers in the pathogenesis of aniridia. Apart from HS2 disruption, the translocation also leads to a positional effect, as it separates *PAX6* from some of its essential cis-enhancers of the DRR and then prevents their binding necessary for *PAX6* transactivation. Indeed, this translocation leads to deregulation and haploinsufficiency of *PAX6,* as we observed decreased expression in patient-derived LCLs similar to other patients carrying disease-causing *PAX6* variants. However, we cannot conclude which is the main contributor to *PAX6* deregulation in our patient, as either HS2 disruption or the positional effect could solely cause it. We speculate whether a more complex synergistic effect of both events is more likely to be involved.

Interestingly, HS2 localizes between E180B and SIMO, two well-characterized and highly conserved enhancers for ocular *PAX6* expression [4, 31], which have also been previously involved in the etiopathogenesis of aniridia [2, 10, 13]. Their deletion has been described as the main responsible for aniridia underlying 3’ enhancer microdeletions, as both enhancers lie within the 245 Kb critical region for *PAX6* regulation proposed by Ansari et al. [13], defined by the overlapping breakpoints of different microdeletions identified by CMA analysis. This same strategy allowed us to further refine this critical region for *PAX6* regulation to 18 Kb, in which E180B is the only known cis-regulatory element involved [10]. Our current work also supports this minimal critical region in intron 9 of *ELP4*, as the translocation breakpoint keeps SIMO intact, and E180 is separated from *PAX6* on chromosome 6.

Our work highlights the importance of refining SVs breakpoints first identified by cytogenetic methods using genome-wide molecular strategies to fully understand the molecular mechanisms contributing to pathogenicity. Without breakpoint refinement by LRS, we could not have assumed that the t(6;11) translocation) found in our patient was the actual genetic cause. Although WGS approaches are already very promising for studying complex SVs, balanced variants involving highly repetitive sequences, such as centromeric regions, can still be overlooked. Our work shows that these types of SVs are particularly challenging to uncover even by LRS techniques due to the low performance of the SV calling algorithms available so far. First, we used three different bioinformatic approaches to detect SVs in long-read WGS, but all failed to detect this translocation (Additional file 1: Fig. S4), similar to our previously short-read WGS analysis. Only a further reanalysis with Sniffles2 successfully detected this translocation efficiently. The main reason for overlooking this translocation resides in the inherent difficulties in mapping chimeric reads encompassing the low-complexity centromeric alpha-satellite sequences on chromosome 6. Despite these shortcomings of short-read sequencing, further manual review of the aligned sequence data from the Illumina short-read WGS analysis revealed the presence of a few chimeric reads at 11p.13 supporting the translocation. However, it remained undetected by available bioinformatics tools (Additional file 1: Fig. S6).

This study provides new insights into the role of balanced SVs as a genetic cause of congenital aniridia, which seems to be a rare molecular mechanism to date, as only a few patients have been identified [6–8, 27, 28]. Therefore, our study suggests that balanced inversions and translocations affecting 11p13 may be somewhat more common in aniridia than anticipated. These types of SVs might have been overlooked in other cohorts of *PAX6*-negative cases after genetic screening by conventional methods, such as genome-wide CMA or targeted NGS analysis, both of which are unable to identify copy number neutral SVs.

## Conclusions

Our study demonstrates the great opportunities of nanopore-based LRS to unravel previously hidden SVs in unsolved cases with a particular phenotype with a strong genetic correlation. We report two cases in which a hidden variant in *PAX6* was suspected but remained unsolved despite being studied for many years with complementary approaches. LRS enabled precise breakpoint mapping of causative balanced chromosomal SVs that directly disrupt *PAX6* or its distant regulatory elements, as we demonstrated functionally. This provides new evidence of the importance of positional effects of enhancers as pathogenic mechanisms leading to *PAX6* deregulation underlying congenital aniridia. Finally, our work highlights the importance of using emerging technologies to improve diagnostic rates, through which we achieved a complete genetic characterization of our cohort of patients with classical aniridia.

## Patients and methods

### Patient and sample collection

Both probands are part of a large cohort of 110 families with classical aniridia referred to the University Hospital Fundación Jiménez Díaz (Madrid, Spain) for *PAX6* testing. Patient-derived lymphocyte cell lines (LCLs) were established by Epstein-Barr virus (EBV)-mediated transformation of blood lymphocytes. High Molecular Weight (HMW) genomic DNA was isolated from peripheral whole blood or LCLs using the Puregene® Blood kit DNA (Qiagen, HILDEN, Germany). Total RNA from LCLs of the two probands, three *PAX6*-mutated patients, and two control individuals was isolated with TRIzol reagent (Invitrogen, Thermo Fisher Scientific, MA, USA), following standard procedures.

### PAX6 screening

Both patients were screened for SNVs and microdeletions in *PAX6* using Sanger sequencing and MLPA (multiplex ligation-dependent probe amplification) according to a standard protocol for genetic analysis of congenital aniridia [23]. Chromosomal analysis was also performed by Giemsa-banding karyotyping on cultured peripheral blood lymphocytes from probands and parents using standard procedures. In addition, CMA was performed probands using array-based comparative genome hybridization (aCGH). ANI-1 was screened using the WAGR array, as previously reported [5]. ANI-2 was screened using a customized aCGH (4 × 180 K, Agilent Technologies, Santa Clara, CA, USA) targeting 150 eye developmental-related genes [10]. Both microarrays covered 5 Mb of the *PAX6 locus* on 11p13 at high-resolution.

### Short-read sequencing

Proband ANI-1 was screened using a custom ANIRIDIA panel using HaloPlex capture (Agilent Technologies) for the full *PAX6* gene (exons, introns, and UTRs), 16 known *PAX6* enhancers, and 120 genes for other congenital ocular malformations, as previously described [10]. CNV analysis was performed with four different tools based on read depth algorithms: CONVADING [32], ExomeDepth [33], CODEX2 [34], and PanelcnMOPS [35].

ANI-2 was screened by short-read WGS using the NEB Next Ultra DNA Library kit (New England BioLabs, Ipswich, MA, USA) for library preparation and then paired-end sequenced (2 × 150 bp) on a Novaseq6000 platform (Illumina, San Diego, CA, USA) at 30 × coverage. Raw sequencing reads were aligned to the GRCh38/hg38 assembly using the BWAv0.7.15 with default parameters. CNV analysis was performed with MANTA [36] using its default parameters. Reads at the potential 6p21.3 and 11p15.5 breakpoints junctions of the translocation were visually inspected using the Integrative Genomics Viewer (IGV) [37] to identify discordantly paired reads with unexpected chromosomal mates.

### Long-read sequencing and data analysis

Whole-genome libraries were prepared using 1 µg of HWM-DNA of > 10 Kb fragments with the Ligation Sequencing Kit SQK-LSK109 (Oxford Nanopore Technologies, ONT, Oxford, UK), following the recommended protocol. DNA fragments were first end-repaired using the Ultra II End-Repair Prep module (New England BioLabs) and then incubated with the NEBNext Quick T4 DNA ligase (New England BioLabs) and 1D adapter mix (ONT). After purification, the library was sequenced on R9.4.1 flowcells (FLO-MIN106D) on a PromethION device (ONT) with at least 30 × coverage.

Bioinformatic analysis of the LRS data is summarized in Additional file 1: Figure S1. Briefly, base calling was performed using Guppy. Raw reads were aligned to the human reference assembly (GRCh38/hg38) using the Minimap2 [38]. SV calling from the aligned BAM files was performed using CuteSV [39], SVIM [40], Sniffles, and Sniffles2 [24, 25]. Sniffles2 was executed in multisample SV calling mode by combining variant calling of 34 samples from the same sequencing batch. To improve SV calling in repetitive regions, the ‘–tandem-repeats' parameter was used in combination with the bed file recommended by Sniffles2 (https://github.com/fritzsedlazeck/Sniffles). Prioritization of likely pathogenic SVs was performed by filtering out SV calls by allele frequency (AF), keeping variants with an AF < 0.2, and batch frequency, keeping unique variants within a cohort of 34 samples. SV calls were visually inspected using IGV and Ribbon [41] visualizers.

### Structural variant validation

Potentially pathogenic SV calls affecting the 11p13 *locus* were further validated by cytogenetic and molecular analysis. Chromosomal breakpoints of the 11p13 inversion were confirmed by PCR with flanking primers designed specifically for the proximal and distal junctions using primer3 and NCBI Primer-Blast (http://www.ncbi.nlm.nih.gov/tools/primer-blast/) (Additional file 1: Table S2). Amplicons were analyzed by electrophoresis on a 2% agarose gel and further sequenced on an ABI3130 sequencer (Thermo Fisher Scientific), following standard procedures. Sequences flanking each breakpoint were analyzed using UCSC BLAT and RepeatMasker to identify repeat elements.

FISH analysis was performed on metaphase chromosomes using commercially available FISH probes for *PAX6* (PAX6 –RE 11p13, 152 kb in 5-ROX (5-Carboxy-X-rhodamine, Empire Genomics, NY, USA) and centromeric probes for chromosome 6 (Vysis CEP6-D6Z1, 6p11.1-q11, Aqua, Abbott Molecular, IL, USA), and chromosome 11 (Vysis CEP11-D11Z1, 11p.11-q11, Aqua, Abbott Molecular), following standard procedures. Metaphases were analyzed under a fluorescence microscope, and images were captured using the ISIS Software (MetaSystems, Germany).

### PAX6 expression analysis

Reverse transcription to synthesize full-length cDNA was conducted from 2.5 μg of total RNA using random primers and the SuperScript IV First Strand cDNA Synthesis Kit (Invitrogen, Thermo Fisher Scientific, MA, USA). The cDNA was fluorometrically quantified in a Qubit™ Flex Fluorometer with the dsDNA HS Assay Kit (Thermo Fisher Scientific, MA, USA). Quantitative analysis of mRNA expression was performed by droplet-digital PCR (ddPCR) using TaqMan® Gene Expression assays for *PAX6* (Hs01088114_m1 *PAX6* VIC, Thermo Fisher Scientific) and the housekeeping *RPLP0* (*Ribosomal 2 protein, large, P0*) gene (Hs99999902_m1 *RPLP0* FAM, Thermo Fisher Scientific). ddPCR assays were performed in at least three technical replicates and were run for each gene using the QX200 ddPCR Supermix (Bio-Rad, Hercules, CA, USA) on the QX200 Droplet Digital PCR System (Bio-Rad), according to the manufacturer’s protocol. Ratios of the absolute quantity of *PAX6* transcripts (expressed as copies/μL) were normalized using the *RPLP0* transcripts as a reference in multiplex PCR assays. The non-parametric Kruskal Wallis test was used to assess the statistical significance between the normalized ratios from different groups: ANI-1, ANI-2, *PAX6*-mutated patients, and control individuals, followed by a Dunn’s test for each multiple pairwise comparison with Bonferroni (adjusted p-value < 0.05).

## Supplementary Information

**Additional file 1.** Supplementary Tables and Figures.

## Data Availability

NGS data supporting the conclusions of this article have been deposited at the European Genome-Phenome Archive (EGA https://www.ebi.ac.uk/ega/home), which is hosted by the EBI and the CGR, under accession number EGAS00001007252.
