# Supplementary material for: Long-read genome sequencing identifies cryptic structural variants in congenital aniridia cases
Source: Hum Genomics. 2023 Jun 2;17:45. doi: 10.1186/s40246-023-00490-8 (PMC10236743; doi:10.1186/s40246-023-00490-8)
Supplement: Supplementary file 1 — Additional file 1. Supplementary Tables and Figures. [file 40246_2023_490_MOESM1_ESM.docx]

**Supplementary Information**

**Long-read genome sequencing identifies cryptic structural variants in congenital aniridia cases.**

Alejandra Damián^1,2^, Gonzalo Núñez-Moreno^1,2,3^, Claire Jubin^4^, Alejandra Tamayo^1,2,5^, Marta Rodríguez de Alba^1,2^, Cristina Villaverde^1,2^, Cédric Fund^4^, Marc Delépine^4,^ Aurélie Leduc^4^, Jean François Deleuze^4^, Pablo Mínguez^1,2,3^, Carmen Ayuso^1,2^, Marta Corton^1,2,*^.

1. Department of Genetics & Genomics, Instituto de Investigación Sanitaria-Fundación Jiménez Díaz University Hospital - Universidad Autónoma de Madrid (IIS-FJD, UAM), Madrid, Spain.

2. Centre for Biomedical Network Research on Rare Diseases (CIBERER), Madrid, Spain.

3. Bioinformatics Unit, Instituto de Investigación Sanitaria-Fundación Jiménez Díaz University Hospital - Universidad Autónoma de Madrid (IIS-FJD, UAM), Madrid, Spain.

4. Centre National de Recherche en Génomique Humaine, Université Paris-Saclay, 91057, Evry, France.

5. Department of Surgery, Medical and Social Sciences, Faculty of Medicine and Health Sciences, Science and Technology Campus, University of Alcalá, 28871 Alcalá de Henares, Spain

***Author for correspondence**

Marta Corton, PhD

Department of Genetics and Genomics.

IIS–Fundación Jiménez Díaz University Hospital

Avenida Reyes Católicos, 2. Madrid 28040, Spain

email address: mcorton@fjd.es

**Supplementary Table S1**. **ONT sequencing quality.**

| Sample ID | Sequenced bases | Reads | Mapping rate  (hg38) | Median length (bp) | Max length  (bp) | N50 (pb) | Median read depth  (bp) | No. uniquely mapped reads | Targeted bases  with coverage ≥10 (%) | Targeted bases  with coverage ≥30 (%) |
| --- | --- | --- | --- | --- | --- | --- | --- | --- | --- | --- |
| ANI-1 | 1,38129E+11 | 19,434,553 | 99.86% | 9971 | 244,415 | 11,730 | 43 | 13,992,453 | 88.07% | 85.16% |
| ANI-2 | 1,26994E+11 | 26,632,886 | 99.31% | 4800 | 427,369 | 12,725 | 39 | 18,529,455 | 88.02% | 79.95% |

**Supplementary Table S2. Specific primers designed for validation of breakpoints of the 11p13 inversion identified**

**in patient ANI-1 by PCR and Sanger sequencing.**

| Name | Junction | Sequence (5’-3’) |
| --- | --- | --- |
| JX1_DNA_F | JX1 fragment | ATGCCTCACTCCACTCCCTA |
| JX1_DNA_R |  | CTGCTGTGAAGCCAGATTTC |
| JX2_DNA_F | JX2 fragment | AATAGGTGTCAAGGCGAAGC |
| JX2_DNA_R |  | TTCCGATGAAAATGCAAAAC |

**Supplementary Table S3**. **Statistical analysis of *PAX6* expression.**

| **.y.** | **group1** | **group2** | **n1** | **n2** | **statistic** | **p** | **p.adj** | **p.adj.signif** |
| --- | --- | --- | --- | --- | --- | --- | --- | --- |
| Ratio | ANI-1 | ANI-2 | 9 | 8 | -0,5608112 | 0,57492622 | 1 | ns |
| Ratio | ANI-1 | Mut-PAX6 | 9 | 14 | 1,36356541 | 0,17270439 | 1 | ns |
| Ratio | ANI-1 | wt-PAX6 | 9 | 19 | 4,7003941 | 2,5966E-06 | 0,00001558 | **** |
| Ratio | ANI-2 | Mut-PAX6 | 8 | 14 | 1,92932972 | 0,05368994 | 0,32213965 | ns |
| Ratio | ANI-2 | wt-PAX6 | 8 | 19 | 5,15946307 | 2,4766E-07 | 1,486E-06 | **** |
| Ratio | Mut-PAX6 | wt-PAX6 | 14 | 19 | 3,74605488 | 0,00017964 | 0,00107782 | ** |

Statistical analysis was performed using the Kruskal–Wallis test with Dunn's test for multiple comparisons of normalized ratios

of *PAX6* expression, and Bonferroni for multiple testing corrections. Significance was defined as *p.adj < 0.05, ** p.adj < 0.01,

**** p.adj < 0.0001 and p.adj >0.05 (not significant, ns).

**
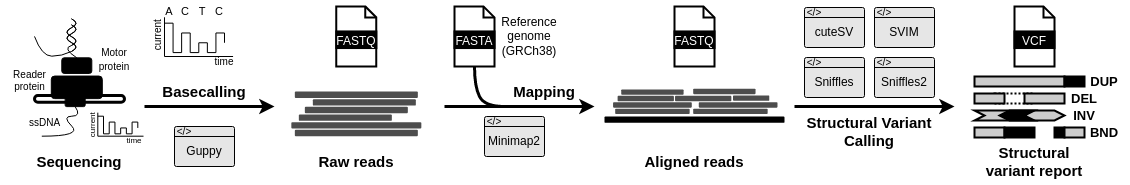
**

**Supplementary Figure S1. Workflow of the nanopore-based long-read sequencing, bioinformatic processing data, and structural variant calling.**

The Nanopore sequencing was performed on R9.4.1 flowcells on a PromethION device (Oxford Nanopore Technologies, ONT) with at least 30x coverage. After sequencing, LRS data was processed following standard procedures of ONT. First, basecalling was performed using Guppy, and the obtained raw reads were aligned to the human reference assembly (GRCh38/hg38) using the aligner Minimap2. From the Minimap3 alignments, SVs calling was performed using four bioinformatic tools: CuteSV, SVIM, Sniffles, and Sniffles2, obtaining an SV report that contained data for duplication (DUP), deletion (DEL), inversion (INV), and break ends (BND) calls.


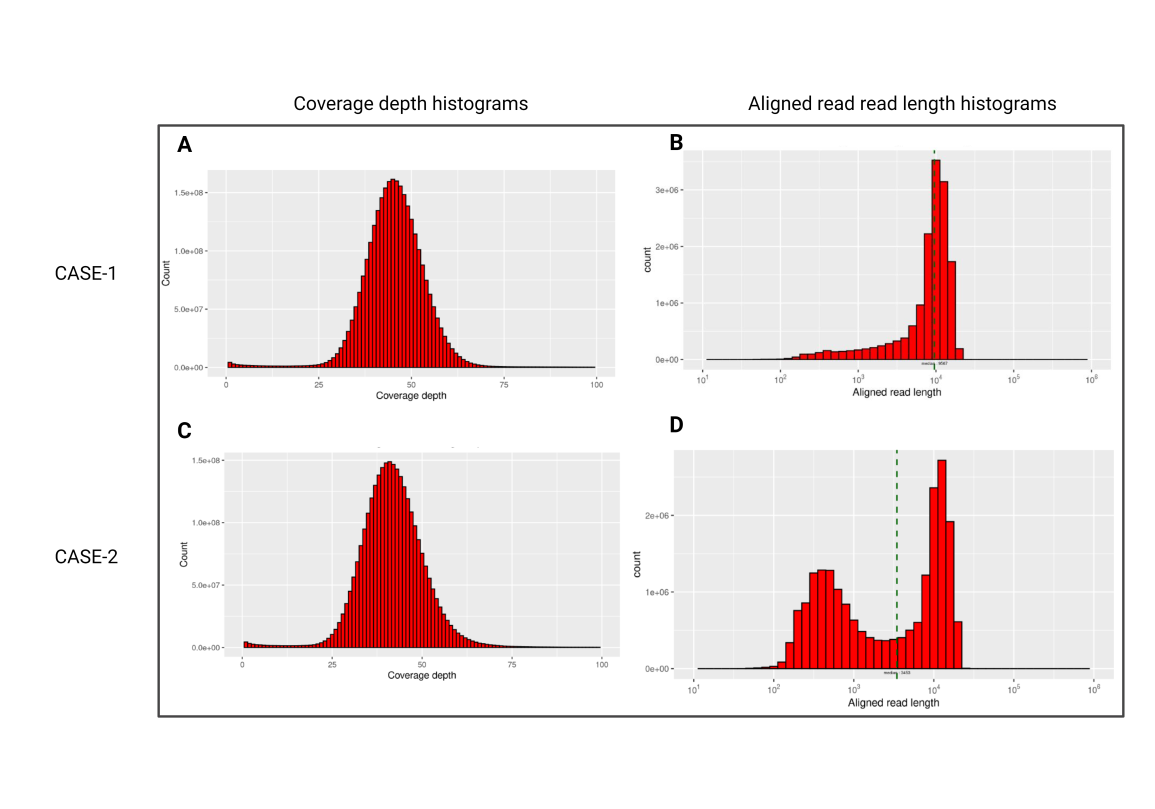


**Supplementary Figure S2. Histograms of coverage depth and read length of long-read Whole genome sequencing.**

**
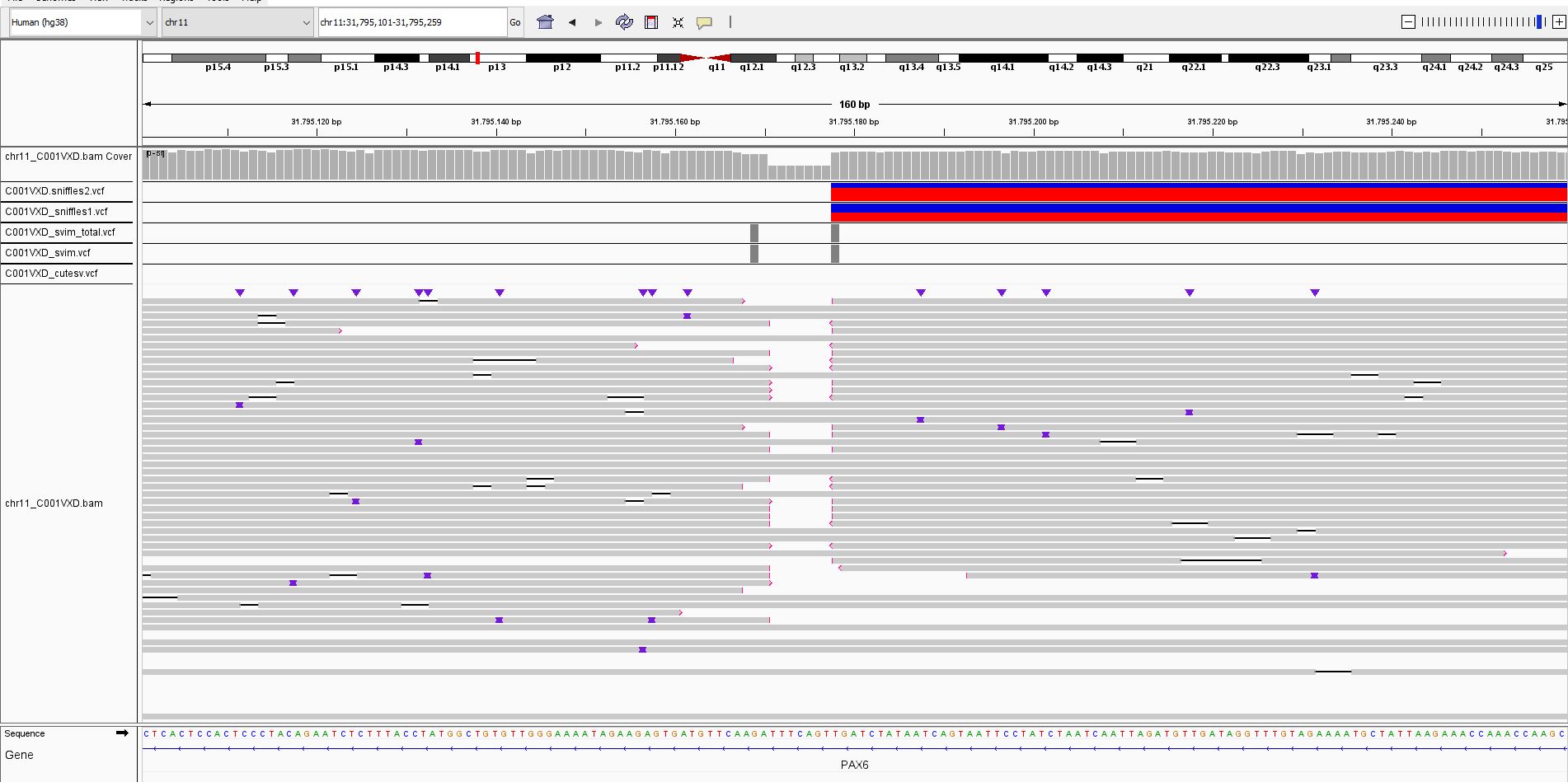
**

**Supplementary Figure S3. SV calling of long-read whole genome sequencing in patient ANI-1.** IGV screenshot representing the aligned long-read alignments in the proximal breakpoint of the 11p13 inversion within the intron 7 of *PAX6*. VCF track with SV calling from Sniffles2, CuteSV, SVIM, and Sniffles1, read coverage, and alignment tracks are shown from top to bottom. Three SV algorithms indicate the presence of a structural variant at this point. SVIM indicated the presence of a breakpoint at chr11:31,795,178 and Sniffles and Sniffles2 revealed the presence of a heterozygous inversion with chimeric reads mapping on 11p12. Sniffles1 and sniffes2 vcf tracks showing variants (allele freq –reference(blue); allele freq-variant (red). Notice: Cutesv did not detect any variant


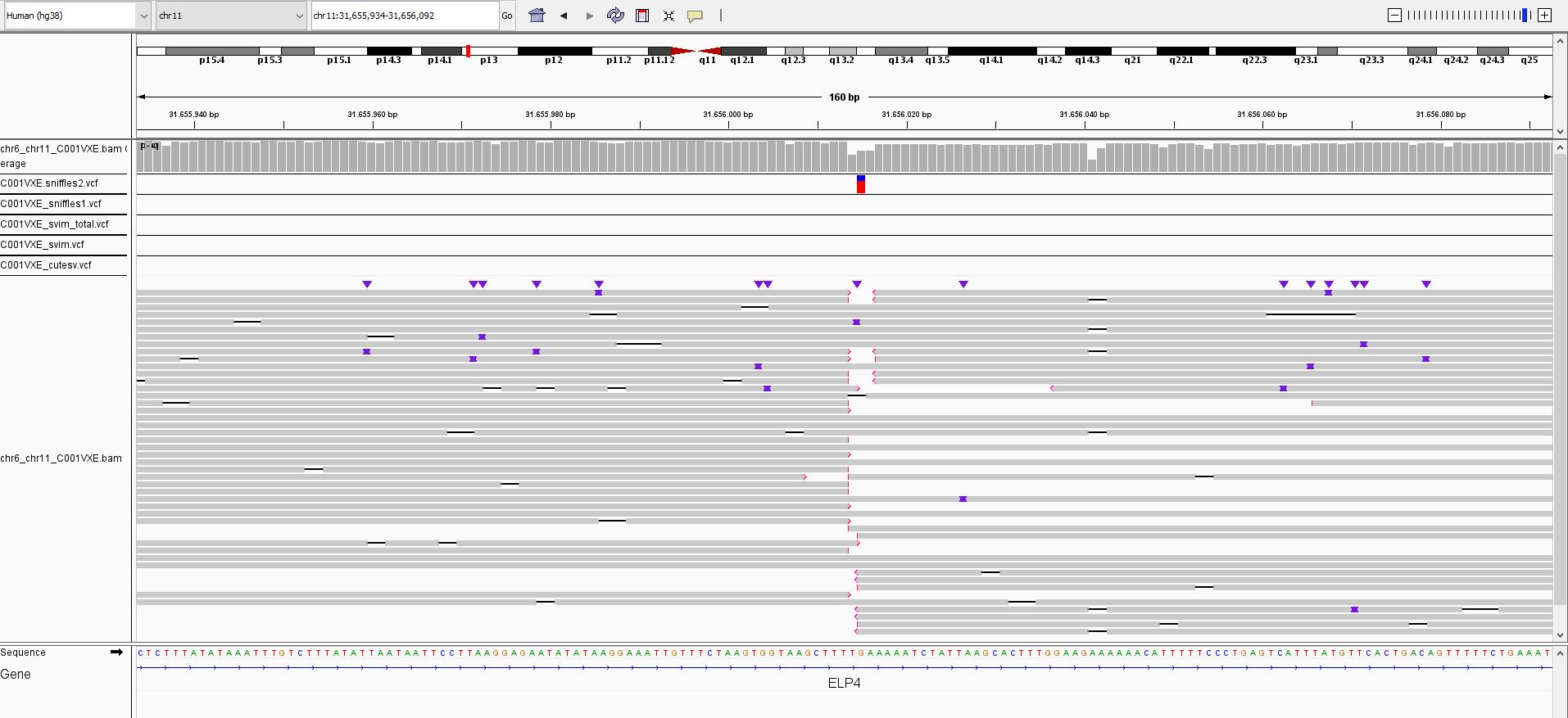


**Supplementary Figure S4. SV calling of long-read whole genome sequencing in patient ANI-2.** VCF track with SV calling from Sniffles2, CuteSV, SVIM and Sniffles, read coverage, and alignment tracks are shown from top to bottom. IGV screenshot representing the aligned long-read alignments in the breakpoint of the translocation t(6,11) in chromosome 11p13. Of the four SV calling algorithms, only Sniffles2_vcf indicates a breakpoint chr11:31,656,015 and revealed the chimeric reads with inter-chromosomal rearrangement with chromosome 6p11.1. (allele freq –reference (blue) and allele freq-variant (red).

**A.**

**B.**


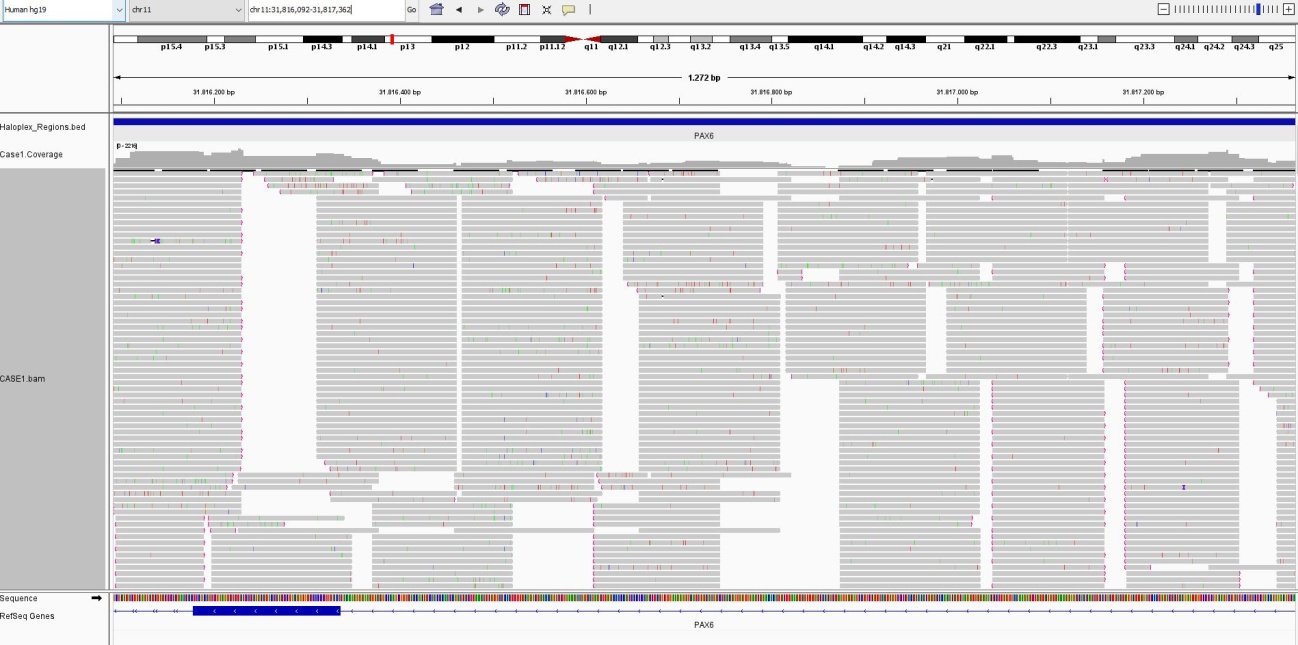


**Supplementary Figure S5. Screening of SVs in intron 7 of *PAX6* in patient ANI-1. A.** Schematic representation of intron 7 reflecting the localization of different elements: the proximal breakpoint of the 11p13 inversion (in red), the putative 451-bp microdeletion (in blue) detected at low confidence by short-read gene panel and the specific amplicons (in grey) and the 60-mer CRH-array probes (in black) used to capture this region using an Haloplex-based capture NGS panel and custom chromosomal array analysis. *PAX6* exons 7 and 8 are represented in cyan blue. **B.** IGV screenshot representing the aligned short-read alignments and coverage in the intron 7 of *PAX6* after capture by Haloplex gene panel. Coverage bed file track (blue).

**A.**


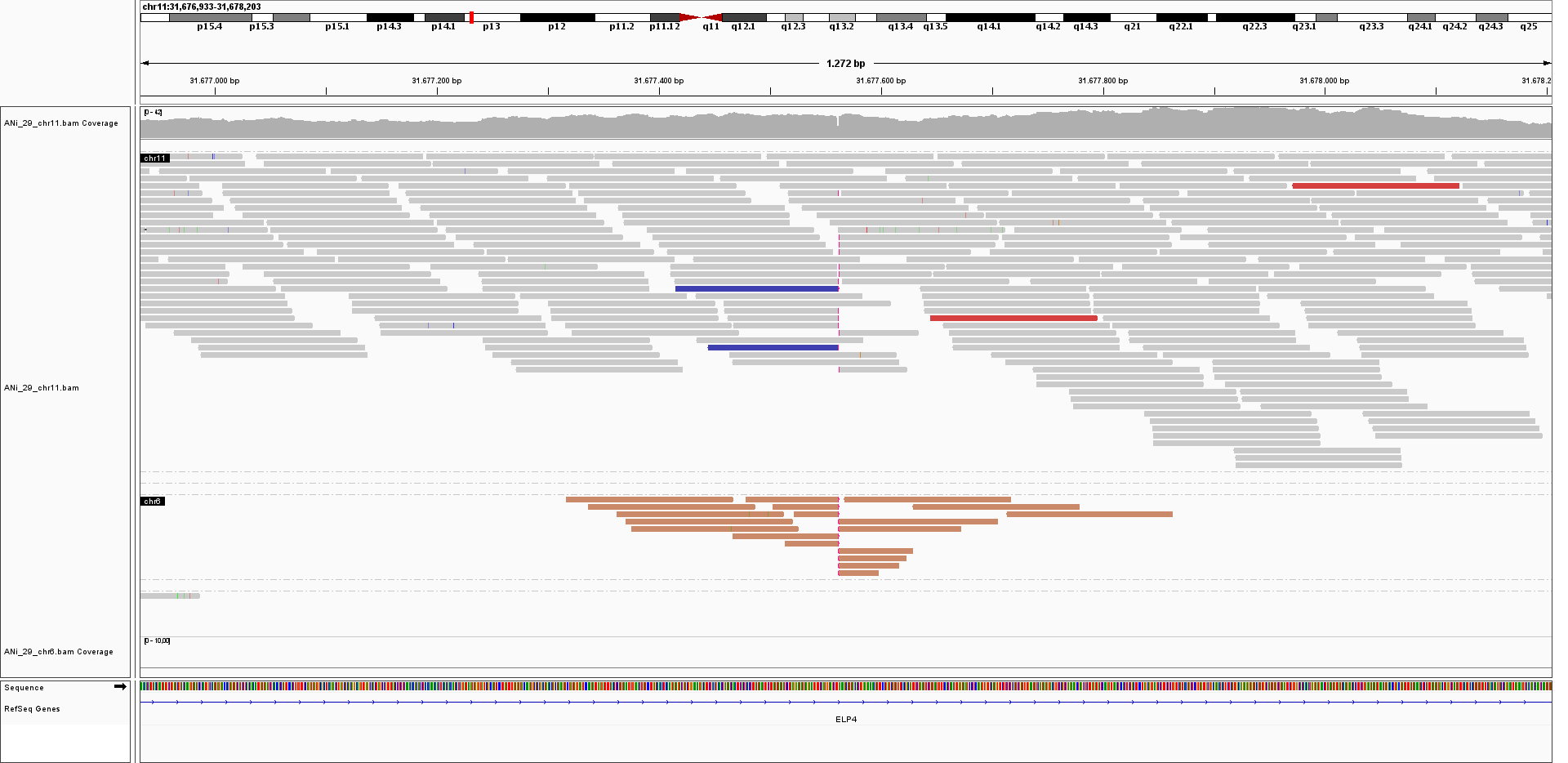


**B.**


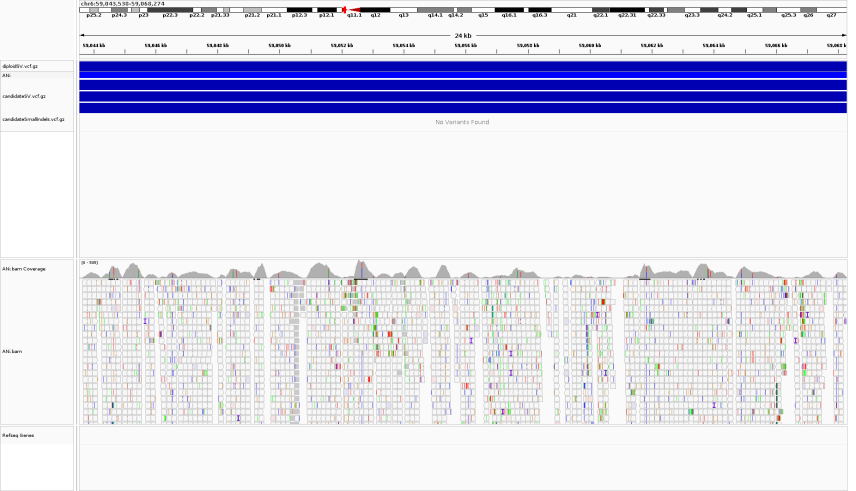
**Supplementary figure S6. SV calling of short-read whole genome sequencing in patient ANI-2.** VCF track with SV calling, read coverage, and alignment tracks are shown from top to bottom. **A.** IGV screenshot representing the aligned short-read alignments in the breakpoint of the translocation t(6,11) in chromosome 11p13. Reads are grouped and colored by insert size and chromosome mate. Normal paired reads are colored in grey. Manual inspection revealed a breakpoint at chr11:31,656,015 (hg38 here lift over at hg19 chr11:31,677,544), which is surrounded by discordant reads colored in brown indicating an inter-chromosomal rearrangement with chromosome 6. **B.** IGV screenshot representing the aligned short-read alignments in the breakpoint of the translocation t(6,11) in chromosome 6p11, showing a low coverage with most of the reads having a mapping quality of zero (reads with light gray borders) due to the highly repetitive nature of the centromeric sequences.
